# Can the Testing Effect for General Knowledge Facts Be Influenced by Distraction due to Divided Attention or Experimentally Induced Anxious Mood?

**DOI:** 10.3389/fpsyg.2019.00969

**Published:** 2019-05-03

**Authors:** Chi-Shing Tse, Meingold Hiu-Ming Chan, Wai-Shing Tse, Savio Wai-Ho Wong

**Affiliations:** ^1^Department of Educational Psychology, The Chinese University of Hong Kong, Shatin, China; ^2^Centre for Learning Sciences and Technologies, The Chinese University of Hong Kong, Shatin, China; ^3^Department of Human Sciences, The Ohio State University, Columbus, OH, United States; ^4^School of Arts and Humanities, Tung Wah College, Hong Kong, China

**Keywords:** testing effect, anxiety, attention, fact learning, working memory

## Abstract

Studies on testing effect have showed that a practice test on study materials leads to better performance in a final test than restudying the materials for the same amount of time. Two experiments were conducted to test how distraction, as triggered by divided attention or experimentally induced anxious mood in the practice phase, could modulate the benefit of testing (vs. restudying) on the learning of interesting and boring general knowledge facts. Two individual difference factors (trait test anxiety and working memory (WM) capacity) were measured. Under divided attention, participants restudied or recalled the missing information in visually presented general knowledge facts, while judging whether auditorily presented items were from a pre-specified category. To experimentally induce anxious mood, we instructed participants to view and interpret negative pictures with anxious music background before and during the practice phase. Immediate and two-day delayed tests were given. Regardless of item type (interesting or boring) or retention interval, the testing effect was not significantly affected by divided (vs. full) attention or anxious (vs. neutral) mood. These results remained unchanged after taking into account the influences of participants’ trait test anxiety and WM capacity. However, when analyses were restricted to the study materials that had been learnt in the divided attention condition while participants accurately responded to the concurrent distracting task, the testing effect was stronger in the divided attention condition than in the full attention condition. Contrary to previous studies (e.g., Tse and Pu, 2012), there was no WM capacity × trait test anxiety interaction in the overall testing effect. Theoretical and practical implications of these findings are discussed.

## Introduction

Testing has often been used to assess how much students have learned in the classroom. Other than the evaluative purpose, studies have reported the testing effect: a practice test on the study materials leads to better performance in a final test than restudying them for the same amount of time. The robust benefit of testing generalized across study materials (e.g., general knowledge fact), test types(e.g., cued recall), and populations (e.g., older adults) (see, e.g., Rowland, 2014; Kornell and Vaughn, 2016; Putnam et al., 2016, for reviews). However, less is known about whether this testing effect could be modulated by situational factors that can be experienced by students in a daily classroom setting (e.g., distraction), as well as the interaction between these situational factors and individual differences such as working memory (WM) capacity and trait test anxiety. In the current study, we manipulated the potential distraction that students may experience (divided attention or anxious mood in the restudying and testing practice phases) to examine how these situational factors would influence the testing effect for the learning of interesting and boring general knowledge facts in immediate and two-day delayed tests. We tested the replicability of Tse and Pu’s (2012) findings on whether WM capacity could interact with trait test anxiety in predicting the testing effects and examined whether these two individual difference factors could interact with situational factors (divided attention or anxious mood) in predicting the testing effects.

### The Effect of Divided Attention on Test-Enhanced Learning

Using older adults as participants, Tse et al. (2010) compared the benefits of testing (vs. restudying) on cued recall of unfamiliar face-name pairs. When there was no corrective feedback in the practice phase, adults aged over 80 years, who showed impaired attention in psychometric tests, did not benefit from testing and even showed a better performance in the restudying than testing condition in the final test. This might show an important role of attention in the practice phase in producing the testing effect (see also Dudukovic et al., 2015, for similar findings in children with attention-deficit hyperactivity disorder). However, other studies directly manipulated the full vs. divided attention in young adults showed conflicting evidence. Under divided attention, individuals have to monitor and process information from multiple sources simultaneously. Dudukovic et al. (2009) and Gaspelin et al. (2013) reported the negative and null effect of divided attention, respectively, on performance in a repeated memory test, although neither of these studies included a restudying condition and thus could not examine the testing effect. In two experiments with a restudying condition, Mulligan and Picklesimer’s (2016) participants studied word pairs, performed restudy/testing practice phase under full or divided attention by a digit classification secondary task, followed by a final cued-recall test. The testing effect was stronger in the divided than full attention condition, regardless of whether the word pairs were related or unrelated, whether there were feedbacks during retrieval practice, whether the final test was given immediately or 1 day after the practice phase, and whether participants’ distracting task performance was high or low. Mulligan and Picklesimer (2016) concluded that the encoding effects of retrieval at testing might be more resilient in the face of distraction than the encoding effects of restudying, thus producing a stronger testing effect in the divided attention than in the full attention condition.

Mulligan and Picklesimer’s (2016) results could be explained by considering the link between attention selection under competitive distraction and memory encoding, as suggested by Santangelo and his colleagues (e.g., Santangelo and Macaluso, 2013; Pedale and Santangelo, 2015; Santangelo, 2015, 2018).^1^ According to Santangelo (2015), the saliency of an object in a natural scene could modulate its encoding (and in turn retrieval) in WM. When processing resources are limited at encoding (e.g., under divided attention), the highly salient objects are prioritized in encoding. This may then reduce the available resources to encode and retrieve other objects with relatively lower saliency in the scene. This is similar to attentional priorities of highly salient objects under high conflict or competition among stimuli (e.g., Bundesen et al., 2011). Given that retrieval practice (vs. restudying) can make the studied information more salient (e.g., Kuo and Hirshman, 1997; Kliegl and Bäuml, 2016), Santangelo and his colleagues’ view would predict that when attention is divided in the practice phase, participants may prioritize the processing of more salient information (i.e., those being retrieved), as opposed to performing the distracting task. On the contrary, less salient information (i.e., those being restudied) might not receive such attentional priorities over the distracting task. As participants might allocate more attentional resources to the items being tested than those being restudied in the practice phase when their attention is divided and this might not be the case when they are under full attention, they are expected to show a stronger testing effect in the divided vs. full attention condition (Hypothesis 1a).

However, there was a limitation in previous studies that might have led to no (or even positive) influence of divided attention on the testing effect. Specifically, their study and distracting materials were quite different in nature [verbal study materials vs. tone-counting (Gaspelin et al., 2013) or digit classification secondary task (Mulligan and Picklesimer, 2016)]. It is noteworthy that dividing attention at retrieval more likely impaired performance when both study and distracting materials were verbal (e.g., Fernandes and Moscovitch, 2003). Thus, the negative impact of divided attention on the testing effect might have been underestimated in those studies. In the current study, we addressed this problem by using verbal study materials and verbal distracting task in the practice phase.^2^ Dividing attention at retrieval could impair performance when both study and distracting materials are verbal, disrupt elaborative processing during retrieval practice (Carpenter, 2009), and in turn reduce the benefit of testing on later memory retrieval. This would then predict that the testing effect might be stronger in the full attention than divided attention condition (Hypothesis 1b), opposite of the prediction derived from Santangelo and his colleagues’ works.

### The Effect of Anxious Mood on Test-Enhanced Learning

Apart from the effect of divided (vs. full) attention on the benefit of testing over restudying, it is important to examine if other types of distraction might compromise the benefit of testing over restudying. Research showed that anxious mood increases students’ susceptibility to distraction from task-irrelevant thoughts and thus impairs their memory (e.g., Cassady, 2004). However, few studies have examined the effect of anxious mood on the benefit of testing over restudying in memory performance. Hinze and Rapp (2014) tested how instruction-induced state anxiety modulated the benefit of testing. They used science texts as study materials and multiple-choice and open-ended application questions in their delayed test. In the retrieval practice phase, they manipulated participants’ state anxiety by inducing performance pressure via offering them bonus money for their high test performance. Those who received this bonus instruction showed higher state anxiety in the practice phase. In the delayed test, these participants showed lower proportion recall (relative to their initial-test performance in the practice phase) than those who did not receive the bonus instruction and also those in the control group who only restudied the materials without any bonus instruction in the practice phase. Thus, performance pressure boosted state anxiety, and in turn minimized the benefit of testing in the delayed test. However, their mood manipulation likely triggered both anxious feeling, a simple arousal and of negative mood, and worry related to performance that could be in a form of thought pre-occupation. It is also noteworthy that Hinze and Rapp intended to compare the benefit of retrieval practice with vs. without performance pressure and did not manipulate participants’ mood in both restudying and testing conditions. Hence, they did not specifically test the influence of anxious mood during the practice phase on the subsequent testing effect (i.e., benefit of testing over restudying).

Unlike Hinze and Rapp (2014), the current study did not trigger participants’ anxious mood by asking them to recall test-relevant experience and pre-occupy them with worrying thought. Instead, we presented participants with music clips and pictures that were found to have successfully induced anxious mood. This allowed us to test whether this mood induction, despite not related to test experience, would trigger participants’ state anxiety, which is a situation-related reaction to a perceived threat but not relevant to their performance, and in turn reduce their testing effect. Similar to the influence of state anxiety, individual with high trait test anxiety, a stable disposition to react with excessive worry and irrelevant thought in evaluative situations (e.g., Zeidner, 1998), might be prompted to have worrying thoughts that could reduce the availability of attentional resources. Given that our mood-induction stimuli did not directly trigger test-relevant experience, the detrimental influence of anxious mood might be less likely to happen. Hence, we expected that the testing effect would only be slightly weaker in the anxious than neutral mood condition (Hypothesis 2).

### Individual Differences in the Testing Effect

Compared with the robust testing effect, much fewer studies have examined the role of participants’ individual differences (e.g., WM capacity) in moderating the testing effect. The evidence for these has been quite mixed in the literature (e.g., Wiklund-Hörnqvist et al., 2014; Agarwal et al., 2016). For example, Agarwal et al. (2016) found that participants with lower WM capacity showed stronger testing effect in the two-day delayed test after they were provided feedback in the practice phase. However, Wiklund-Hörnqvist et al. (2014) did not find any relationship between WM capacity and testing effect (see also Minear et al., 2018). Tse and Pu (2012) investigated how the testing effect on Swahili-English word-pair learning in a one-week delayed test was modulated by participants’ WM capacity and trait test anxiety. They reported an interaction between trait test anxiety and WM capacity: testing effect decreased with trait test anxiety for participants with lower WM capacity, but it did not correlate with trait test anxiety for those with higher WM capacity. However, neither Swahili nor English was the native language for their Chinese-speaking participants, so it is unclear whether their results could be merely due to participants’ anxiety toward foreign language learning. Nevertheless, we followed Tse and Pu’s finding and predicted that there would be an interaction between trait test anxiety and WM capacity on the testing effect (Hypothesis 3).

### Potential Role of Working Memory Capacity and Trait Test Anxiety in the Influence of Distraction on the Testing Effect

Individual differences in WM capacity and trait test anxiety could play roles in altering the effect of distraction (divided attention and induced anxiety) on the benefit of testing. Top-down modulation in WM influences one’s ability to prioritize the information processing under competitive distraction (Gazzaley and Nobre, 2012). Given that (a) attentional resource and WM utilize a common, limited pool of processing resource (e.g., Santangelo and Macaluso, 2013) and (b) attentional resource should be allocated to more than one task under divided attention, participants with smaller WM capacity might be more susceptible to the influence of divided attention on the testing effect, which could lead to a stronger testing effect (based on Santangelo, 2015; Mulligan and Picklesimer, 2016) or a weaker testing effect (based on Dudukovic et al., 2009). Some studies showed that participants with higher trait test anxiety were more sensitive to the influence of stressful situation (which might induce state anxiety) on memory performance (e.g., Sorg and Whitney, 1992), whereas others showed that the effect of state anxiety on memory could be independent of one’s trait anxiety (e.g., Harris and Cumming, 2003). To our knowledge, no testing-effect study has examined the interactive effects among WM capacity, trait test anxiety, and divided attention (or anxious mood) manipulation.

### Present Study

Only few studies have examined the situational factors that modulated the benefit of testing over restudying and they reported mixed results (e.g., Dudukovic et al., 2009. vs. Mulligan and Picklesimer, 2016) or had some methodological concerns that might cloud the interpretation of the findings (e.g., Tse and Pu, 2012). The overarching goal of our current experiments was to examine whether the benefit of testing (vs. restudying) in the learning of general knowledge facts, which were presented in young-adult participants’ first language (Chinese), could be affected by the distraction during the practice phase. The distraction was triggered by divided attention via performing a verbal distracting task (Experiment 1) or by anxious mood as induced by a music + picture mood induction procedure (Experiment 2).

The effect of divided attention on the benefit of testing over restudying is unclear. Some studies (e.g., Mulligan and Picklesimer, 2016, see also Santangelo, 2015) might suggest that the testing effect would be stronger in the divided attention than in the full attention condition (Hypothesis 1a), but other studies (e.g., Dudukovic et al., 2009) might suggest that the testing effect would be stronger in the full attention than in the divided attention condition (Hypothesis 1b). The testing effect was expected to be slightly stronger in the neutral mood than in the anxious mood condition (Hypothesis 2). Previous studies reported that item type (e.g., difficulty) could have an effect on the benefit of testing over restudying (e.g., Minear et al., 2018). To test whether the influence of divided attention or anxious mood on the testing effect could be generalized across learning materials, we manipulated the interestingness of the facts (hereafter item type). Apart from the manipulated variables (attention/mood, item type, and practice mode: restudying vs. testing, retention interval), we measured participants’ WM capacity and trait test anxiety as individual difference factors to test whether Tse and Pu (2012)’s interactive effect of WM capacity and trait test anxiety would be replicable with study materials that did not involve foreign language learning (Hypothesis 3). Moreover, we wanted to verify whether the effect of divided attention or anxious mood would still occur after taking into account these individual difference factors. It was not clear how the effect of testing (vs. restudying) would be influenced by any one of these (or their interacting) factors, so we explored the possibilities of these interaction effects without any specific hypotheses.

## General Method

### Participants

Chinese-English bilingual undergraduates participated in exchange for HK$150. Informed consent was sought at the beginning of the study. All experiments were approved by Survey and Behavioral Research Ethics Committee in the first author’s institution.

### Materials

We conducted a norming study on 433 general knowledge facts selected from Holmes (2012); Tauber et al. (2013), and http://www.hk-place.com/, to identify the interesting and boring facts. Facts written in English were translated in Chinese and back-translated to English to ensure that they shared the same meaning as in the original source. For each fact, an answer (mostly nouns) was identified. For each fact on 7-point scales (7 = highest), 28 participants were recruited to rate familiarity, interestingness, and ease of learning, and another 28 participants were recruited to rate familiarity, valence, and arousal. The designated answers of the facts were not highlighted during these rating tasks. The rating tasks were blocked based on the scales, with all 433 facts being randomly presented within each block. The familiarity block was always presented first, with the presentation order of the ease of learning and interestingness blocks (or the valence and arousal blocks) being counterbalanced across participants. We did so to ensure that participants’ familiarity ratings were based on their prior knowledge about the facts, rather than potential priming effects due to exposure within the rating task. Both participant groups’ familiarity ratings were strongly correlated (*r* = 0.91) and yielded equal inter-rater reliability, so we averaged their familiarity ratings for stimulus selection.

We first used interestingness of 4 as the cutoff to categorize the interesting facts (above 4; 199 out of 433) and boring facts (below 4; 234 out of 433). Given its moderate-to-strong correlation with ease of learning (+0.48, *p* < 0.01) and arousal ratings (+0.78, *p* < 0.01), it was quite impossible to control for the latter two variables. Hence, we chose one set of facts with high interestingness, ease of learning, and arousal ratings as interesting facts (e.g., about 1.2 million mosquitoes are needed to absorb all the blood in an adult), and the other with low interestingness, ease of learning, and arousal ratings as boring facts (e.g., the term “first aid” appeared in the United Kingdom as early as 1878), while matching on the two groups’ valence, familiarity, number of characters in the facts, and number of characters in the answers (see Table 1). The match in familiarity ensured that participants’ prior knowledge on the interesting and boring facts should be comparable. Neutral facts (medium in valence) were used as anxious-mood participants remember emotional information better than neutral-mood ones (e.g., MacLeod, 1990; Kensinger, 2009). While the interesting and boring facts did differ in ease of learning and arousal, interestingness still significantly differed between the two groups after controlling for these two variables, *F*(1,76) = 69.13, *MSE* = 0.13, *p* < 0.01, and $\eta_{p}^{2}$ = 0.48. The arousal no longer significantly differed after controlling for interestingness and ease of learning, *F*(1,76) = 0.23, *MSE* = 0.07, *p* = 0.64, and $\eta_{p}^{2}$ = 0.003. While ease of learning did differ significantly after controlling for arousal and interestingness, *F*(1,76) = 4.48, *MSE* = 0.18, *p* < 0.05, and $\eta_{p}^{2}$ = 0.06, its effect size (0.06) was much smaller than the above analyses for difference in interestingness (0.48). Hence, it is reasonable to label the two groups of facts as interesting vs. boring facts.

**Table 1:** Characteristics of general knowledge facts.

	Inter-rater reliability (in Cronbach’s α)	Boring facts	Interesting facts	*p*
Interestingness	0.89	3.01 (0.42) {2.04,3.68}	4.85 (0.45) {4.32,5.86}	<0.01
Ease of learning	0.82	3.51 (0.40) {2.57,4.82}	4.46 (0.48) {3.46,5.32}	<0.01
Valence	0.70	4.22 (0.25) {3.43,4.86}	4.09 (0.46) {3.21,4.93}	0.12
Arousal	0.79	3.19 (0.21) {3.00,4.00}	3.99 (0.41) {3.11,4.86}	<0.01
Familiarity	0.92	2.46 (0.23) {2.09,2.93}	2.38 (0.25) {1.75,2.86}	0.13
Number of characters in the facts	N/A	27.03 (9.10) {12,47}	26.28 (8.96) {10,45}	0.71
Number of characters in the answers	N/A	2.73 (0.68) {1,4}	2.48 (0.72) {2,5}	0.11


**The *p* column indicates the significance level of the difference between the interesting and boring facts. The value in the bracket indicates the standard deviation of the variable. The values in the curly bracket indicate the minimum and maximum values of the variable.**

Apart from the main testing-effect task (see section “Procedures”), all participants performed the following tasks, except that state anxiety scale was given only in Experiment 2 where participants’ mood was manipulated.

#### Test Anxiety Inventory

We used Chinese version of 20-item Test Anxiety Inventory (TAI, Yue, 1996) in 4-point scale to measure trait test anxiety. Participants rated the frequency with which they experience specific symptoms of anxiety before, during and after a typical test. The Cronbach’s α was 0.91 and 0.93 in Experiments 1 and 2, respectively. The TAI score was measured at the beginning of the whole experiment, such that participants’ responses were unlikely contaminated by potential state anxiety being induced during the fact learning.

#### State Anxiety Scale of State-Trait Anxiety Inventory

This scale (STAI-S, Spielberger et al., 1970) is a 20-item inventory in 4-point scale to assess mood at present moment (e.g., I feel worried). We separately summed up the scores based on positive and negative subscales (see Vigneau and Cormier, 2008. for a discussion of this approach). The Cronbach’s αs averaged across the administration at five time points (see below) were 0.93 (*SD* = 0.02) and 0.93 (*SD* = 0.01) for scores of the positive and negative subscales, respectively.

#### Operation Span Task

The automated operation span task shows good internal consistency (0.78) and test-retest reliability (0.83) (Unsworth et al., 2005). In each trial, participants saw a series of letters, each of which was followed by a two-operator arithmetic problem. Across trials, the number of letters for memorization varied randomly from 2 to 7. After each letter-arithmetic-problem sequence, participants recalled letters in the same order as appeared before. High scores are achieved by remembering more letters, while maintaining pre-specified accuracy (>85%) in the arithmetic task. This task was given at the end of the whole experiment to ensure that participants’ WM capacity was minimally influenced by divided attention or anxious mood manipulation. Following Tse and Pu (2012), we used participants’ absolute scores as their WM scores.

### Procedures

PC-compatible computer was used to present stimuli and collect data. Participants were individually tested in a cubicle. The main testing-effect task consisted of study phase, practice phase, immediate test, and delayed test. At the beginning of the study, participants were informed of the procedure in the practice phase.

The 80 general knowledge facts were divided into two sets, each of which consisted of 20 interesting facts and 20 boring facts. One set (40 facts) was assigned as the restudying block, whereas the other, as the testing block. This manipulation was counterbalanced between participants. The facts of these two sets were presented in block, with the presentation order of the two blocks being counterbalanced between participants. Thus, practice mode (testing or restudying) was manipulated within participants. All words of the facts appeared in white DFKai-SB regular font (28 point) on a black background. In the study phase, all facts were presented one at a time for about 11 s. Then, a 6-minute filler task (valence and arousal rating tasks for basic colors) was given in Experiment 1. (This interval was used for mood induction in Experiment 2). In the practice phase, there were two cycles. Previous studies (e.g., Chan and McDermott, 2007; Pu and Tse, 2014) demonstrated the testing effect with two practice cycles. In each cycle, a block of 20 interesting and 20 boring facts appeared as restudying trials, where each fact was presented for 11 s. The designated answers appeared in white bold Microsoft JhengHei font (28 point) to ensure participants focused on the same information in both restudying and testing trials. Another block of 20 interesting and 20 boring facts appeared as testing trials, where each fact was presented for 8 s (plus 3 s feedback, see below). The designated answers were replaced by two underlines, regardless of the number of words in the answers to avoid participants from guessing the answers based on the length of the underline. Participants were instructed to read aloud the answer within 8 s or skip if they failed to recall an answer. Their responses were recorded by the experimenter. After 8 s, regardless of participants’ responses, they saw the correct answer (along with other words of the fact) for 3 s as feedback in the testing trials. In other words, for restudying trials the answer was presented along with other words of the fact for 11 s, whereas for testing trials participants were asked to come up with the answer for the fact within 8 s and then they were given feedback (i.e., the answer presented along with other words of the fact) for 3 s. Both restudying and testing trials lasted for 11 s. The second cycle’s procedure was the same as the first one. After the two cycles, participants received an immediate test where all 80 facts appeared in a similar manner as the testing trials in the practice phase. After 2 days, they were given a delayed test, with the procedure being identical to the immediate test.

## Experiment 1: Effect of Full Vs. Divided Attention on Test-Enhanced Learning

### Methods

#### Sample Size Determination

We conducted *a priori* power analysis by using G^∗^Power, with power = 0.99, alpha = 0.05, number of groups = 2, number of measurements = 2, correlation among repeated measures = 0.50, and non-sphericity correction = 1. We used the smallest effect size in Mulligan and Picklesimer (2016)—partial eta-squared (0.07) for the attention (full or divided) × practice mode (restudying or testing) interaction effect (As stated in Footnote 1, we were not aware of Buchin and Mulligan, 2017, prior to Experiment 1’s data collection even though the attention manipulation of that study was indeed more closely similar to the current experiment than Mulligan and Picklesimer). Based on this power analysis, we needed to recruit at least 32 participants in each of the two conditions (full vs. divided attention) to achieve the statistical power of 0.99. As we intended to control for individual differences (e.g., trait test anxiety) in multiple regression analyses, we recruited up to 48 participants per condition.

#### Participants and Design

A 2 (practice mode, within participants: restudying or testing) × 2 (attention, between participants: full or divided) mixed-factor design was used. Forty-eight participants were recruited for each between-subject condition (see Table 2 for their age and male: female ratio information).

**Table 2:** Experiment 1’s cell means and standard deviation (in parentheses).

	Full attention	Divided attention
Participants’ age	19.81 (1.54)	19.40 (1.51)
Participants’ male: female ratio	24:24	24:24
Proportion correct for interesting facts in the…		
Testing condition in the first/second practice cycle	0.70 (0.15)/0.92 (0.08)	0.67 (0.11)/0.85 (0.12)
Restudying/Testing condition in the immediate test	0.94 (0.06)/0.97 (0.05)	0.88 (0.11)/0.93 (0.08)
Restudying/Testing condition in the delayed test	0.91 (0.10)/0.94 (0.09)	0.86 (0.14)/0.91 (0.09)
Proportion correct for boring facts in the…		
Testing condition in the first/second practice cycle	0.44 (0.15)/0.76 (0.13)	0.37 (0.18)/0.63 (0.18)
Restudying/Testing condition in the immediate test	0.81 (0.15)/0.88 (0.12)	0.68 (0.18)/0.79 (0.16)
Restudying/Testing condition in the delayed test	0.80 (0.16)/0.88 (0.13)	0.69 (0.18)/0.77 (0.18)
WM score	50.17 (15.22)	42.27 (13.64)
TAI score	43.73 (10.23)	46.90 (11.05)


### Procedures

The full and divided attention conditions followed the same procedure as in General Method, except that in the practice phase, participants in the divided attention condition performed the restudying and testing trials concurrently with a distracting task. We adapted Fernandes and Moscovitch’s (2003) word-based distracting task. The distracting items were presented through headphones. The category name was presented at the 1st second and the two exemplars were presented at the 4th and 8th second, respectively. Participants judged whether two auditorily presented items represent concepts under a pre-specified category (e.g., apple in fruit category) by pressing 1 or 2 numeric key to indicate “yes” and “no” responses, respectively. The accuracy of this distracting task was recorded. Following Fernandes and Moscovitch, participants were instructed to perform both restudy/testing and category judgment tasks equally well. Eighty categories were chosen from Chinese young adults’ category norm (Yoon et al., 2004). For each category, we selected 4 exemplars with low-to-moderate typicality to ensure the task difficulty. The 80 categories were divided into two sets: Set A and Set B. For each category in Set A, we selected 4 non-exemplars from four different categories in Set B. Similarly, for each category in Set B, we chose 4 non-exemplars from four different categories in Set A. For half of the participants, categories in Set A were used as the target categories and the exemplars of those in Set B were all non-exemplars, whereas for the other half, categories in Set B were used as the target categories and the exemplars of those in Set A were all non-exemplars. With 4 exemplars and 4 non-exemplars, we created four types of exemplar pairs: two exemplars, exemplar first and non-exemplar second, non-exemplar first and exemplar second, and two non-exemplars. These four pairs were randomly assigned to four general knowledge facts for their restudy/testing trials. There were 80 facts × 2 practice cycles = 160 restudy/testing trials and thus four exemplars from 40 categories in one set (and four non-exemplars from 40 other categories in another set) were used, with all exemplars presented only once for each participant in the experiment.

### Results

We used partial eta-squared and Cohen’s *d* (hereafter *d*, computed using Lakens’, 2013 suggested formula) to indicate the effect sizes of *F* and *t* statistics, respectively. The WM and TAI scores were not correlated (*r* = -0.10, *p* = 0.33). The cell means are reported in Table 2. As there was a significant difference in WM score between participants in the full and divided attention conditions, *t*(94) = 2.68, *p* < 0.01, and *d* = 0.55, but not in TAI scores, *t*(94) = 1.46, *p* = 0.15, and *d* = 0.30, the WM score was included as a covariate in the following analyses. In the divided attention condition, participants’ accuracy of distracting task was lower in the testing condition than in the restudying condition, proportion correct = 0.80 vs 0.90, *t*(47) = 9.65, *p* < 0.01, and *d* = 1.39. Consistent with Santangelo (2015), under divided attention more salient items (i.e., those being retrieved) were likely to receive more attentional resource as compared with the distracting task, whereas less salient items (i.e., those being restudied) were likely to receive as much attentional resource as the distracting task. This explains why participants showed lower distracting-task performance in the testing condition than in the restudying condition.

The participants in the full attention condition performed better than those in the divided attention condition in the first practice cycle differed in boring facts, *t*(94) = 2.20, *p* < 0.05, and *d* = 0.45, but not in interesting facts, *t*(94) = 0.97, *p* = 0.34, and *d* = 0.20. However, the performance in the second practice cycle differed between the two conditions in both interesting and boring facts, *t*(94) = 3.84, *p* < 0.01, *d* = 0.78 and *t*(94) = 3.58, *p* < 0.01, *d* = 0.73, respectively. This stands in contrast to the weak effect of divided attention on memory retrieval in previous studies (e.g., Mulligan and Picklesimer, 2016), which might be due to their divided-attention manipulation not involving verbal materials for both study and distracting tasks. This shows the effectiveness of our divided attention manipulation in the practice phase.

#### Does Divided Attention Modulate the Testing Effect?

Participants’ mean proportion correct in the final tests was submitted to a 2 (practice mode) × 2 (retention interval) × 2 (item type) × 2 (attention) mixed-factor ANCOVA (see “Appendix A” for all statistics). All but attention were within-subject variables, whereas participants’ WM score was included as a covariate. The main effect of attention suggested that participants showed worse performance in divided than full attention condition (proportion correct = 0.81 vs 0.89) [*F*(1,93) = 9.60, *MSE* = 0.08, *p* < 0.01, and $\eta_{p}^{2}$ = 0.09]. The main effect of item type suggested that participants’ performed better for interesting facts (0.92) than boring facts (0.79) [*F*(1,93) = 45.21, *MSE* = 0.02, *p* < 0.01, and $\eta_{p}^{2}$ = 0.33]. The main effect of practice mode demonstrated an overall testing effect (difference of proportion correct = 0.06; 0.88 for testing condition vs 0.82 for restudying condition) [*F*(1,93) = 12.21, *MSE* = 0.01, *p* < 0.01, and $\eta_{p}^{2}$ = 0.12].

**FIGURE 1:**
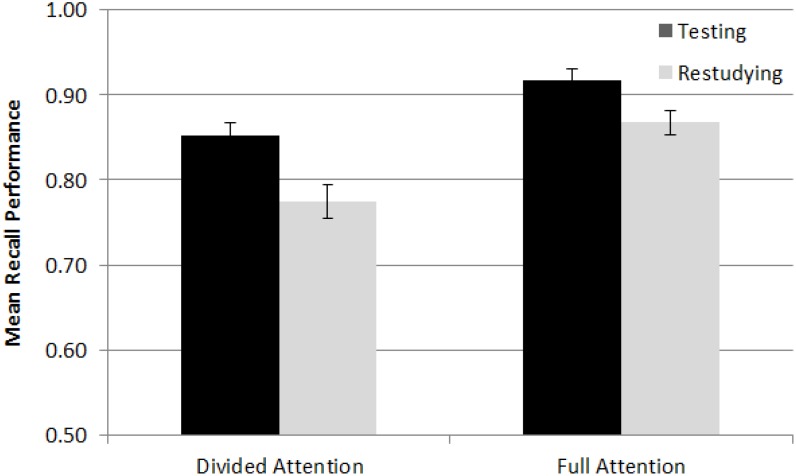
Mean recall performance in the testing and restudying condition of the full and divided attention conditions in Experiment 1. The cell means were averaged across retention intervals (immediate and delayed) and across item types (interesting and boring facts). Error bars indicate the standard errors of means.

As indicated by item type × practice mode interaction, the testing effect was stronger for boring facts (0.09) than interesting facts (0.04) [*F*(1,93) = 6.03, *MSE* = 0.01, *p* = 0.02, and $\eta_{p}^{2}$ = 0.06]. The item type × attention interaction showed that the effect of divided vs. full attention on proportion correct was stronger for boring facts (0.73 vs 0.84) than interesting facts (0.89 vs 0.94) [*F*(1,93) = 6.24, *MSE* = 0.02, *p* = 0.01, and $\eta_{p}^{2}$ = 0.06]. There was no practice mode × attention interaction [*F*(1,93) = 1.96, *MSE* = 0.01, *p* = 0.17, and $\eta_{p}^{2}$ = 0.02] (or any higher-order interaction associated with them), indicating that divided attention did not modulate the testing effect (see Figure 1). Indeed, the testing effect was numerically stronger in the divided attention condition (0.08) than in the full attention condition (0.05). This was consistent, at least with the trend, with Hypothesis 1a, but not with Hypothesis 1b. Nevertheless, three additional analyses are worth considering before drawing a conclusion based on these findings.

First, Dudukovic et al. (2009) reported that dividing participants’ attention during the practice test reduced their memory performance in the subsequent test, even for items that had been correctly remembered in the practice test. To test this possibility, we selected the facts that had been successfully recalled in both tests in the practice phase to represent the testing condition, whereas the facts in the restudying condition were the same as those in the above analyses. Then, we re-ran the analyses on participants’ performance for these facts in the final test. We obtained a significant practice mode × attention interaction [*F*(1,93) = 9.71, *MSE* = 0.02, *p* < 0.01, and $\eta_{p}^{2}$ = 0.10] that the testing effect was significantly stronger in the divided attention (0.20) than in the full attention condition (0.12). This latter finding echoed Mulligan and Picklesimer’s (2016) view that in the practice phase retrieving the facts successfully in the face of distraction could strengthen their memory more than merely restudying them, thus producing a stronger testing effect in the divided (vs. full) attention condition in the final test.

Second, participants’ performance in the distracting task was better in the restudying condition than in the testing condition. The worse distracting-task performance in the testing condition suggested that participants might be more likely to switch attention to process the general knowledge facts when attention was being divided in the testing condition than in the restudying condition. Such enhanced processing due to more attentional allocation might then boost participants’ final-test performance for the facts in the testing condition. In other words, the effect of divided attention might have been underestimated if we included the facts that participants restudied or recalled in the practice phase while they were not responding correctly to the distracting task; that is, when the divided-attention manipulation did not work as expected. To verify this possibility, we selected only the facts that had been learnt when participants in the divided attention condition accurately responded to the distracting task and re-ran the analyses on participants’ performance on these facts. After restricting our analyses on the facts that had been practiced when the divided-attention manipulation worked as expected, there was now a significant practice mode × attention interaction, *F*(1,93) = 4.13, *MSE* = 0.02, *p* = 0.045, and $\eta_{p}^{2}$ = 0.04, showing that the testing effect was stronger in the divided attention (0.09) than in the full attention condition (0.05). This was in line with the findings of Buchin and Mulligan (2017, see also Mulligan and Picklesimer, 2016).

Finally, given that participants were instructed to perform both restudy/testing trials and category judgment tasks equally well, one could question whether all participants were indeed distracted in the practice phase. To test this, we followed a reviewer’s suggestion and computed, for each of the participants in the divided attention condition, the correlation between performance of the category recognition distracting task and performance of testing trials in the practice phase. A positive correlation means that the category recognition task might not be as distracting as we expected, a null correlation means that the category recognition task is indeed distracting, and a negative correlation means that there may be a tradeoff between participants’ choices of performing either task. Across 48 participants, the mean correlation (in kendall tau) was significantly different from zero (+0.31, *p* < 0.01), suggesting that the category recognition might not be as distracting as we expected.

We further examined whether those who showed no significant correlation (i.e., indeed being distracted), relative to those who showed significant positive correlation (i.e., not being distracted), might show a stronger effect of divided attention. Specifically, we classified participants in the divided attention condition based on the significance of their kendall tau correlation (*N* = 19 showed no significant correlation vs. *N* = 29 showed significant positive correlation) and treated this as a “being distracted” between-participant variable. Then, we conducted a mixed-factor ANCOVA (item type × “being distracted” × practice mode × retention interval, with WM score being the covariate). We could not include the attention variable, i.e., full vs. divided attention, in this analysis because the kendall tau correlation (which is used to create the “being distracted” variable) could only be obtained in the divided attention condition. There was a significant main effect of “being distracted,” *F*(1,45) = 4.60, *MSE* = 0.09, *p* < 0.05, and $\eta_{p}^{2}$ = 0.09, indicating that overall performance in the final test was higher for participants who showed positive correlation (i.e., not being distracted) than those who showed no significant correlation (i.e., being distracted; 0.85 vs 0.76, respectively). However, none of the interaction effects associated with this “being distracted” variable was significant, all *F*s < 2.61, *p*s > 0.11, indicating that whether or not participants were distracted (as reflected by the significance of kendall tau correlation) did not modulate the testing effect. We further compared the testing effect between the participants in the full attention condition and those who showed no correlation (i.e., being distracted) in the divided attention condition. The testing effect was marginally larger in the latter group (0.09) than in the former group [0.05, *F*(1,64) = 2.81, *MSE* = 0.01, *p* = 0.098, and η*_p_*^2^ = 0.04], similar to our findings in the omnibus analyses, which were based on all participants with all trials in the divided attention condition. The positive influence of divided attention, despite marginal, on the testing effect provided weak evidence for Hypothesis 1a, but not Hypothesis 1b. To address the potential concern that the group split based on kendall correlation significance might reduce the statistical power to detect the genuine pattern, we ran correlational analyses on the relationship between participants’ kendall tau correlation and their testing effects in various conditions. None of these correlations approached significance (all |*r*|*s* < 0.11, *ps* > 0.46), showing that the magnitude of the testing effect was not significantly associated with the extent to which participants were distracted (as reflected by the magnitude of kendall tau correlation).

#### Do Individual Difference Factors Modulate the Influence of Divided Attention on the Testing Effect?

To explore whether individual difference factors like WM capacity and trait test anxiety might modulate the effect of attention on the benefit of testing, we conducted multiple regression analyses. The testing effect was the dependent variable. The attention variable was coded as +0.5 for full attention and -0.5 for divided attention condition. WM and TAI scores were mean-centered before they were multiplied with the other variables to be interaction terms. In the first step of regression models, we entered participants’ proportion correct in the second practice cycle to control for the extent to which the facts had been learnt varied across participants with different WM and TAI scores. We also entered attention variable and mean-centered TAI and WM scores. Then, three two-way interaction terms and the three-way interaction term were entered in the second and third step, respectively (see “Appendix B”). These interaction terms were added to test whether the influence of attention as a situational factor on the testing effect might be modulated by individual difference factors like participants’ WM, TAI, and their interactive effect. The inclusion of WM × TAI interaction term tested Hypothesis 3—whether Tse and Pu’s (2012) findings could be replicated. There was no problem of multicollinearity, as indicated by a high tolerance (all>0.80). We separately ran the regression analyses for each of the four conditions (interesting facts in the immediate test, interesting facts in the delayed test, boring facts in the immediate test, and boring facts in the delayed test). The results of the full regression models are reported in “Appendix B” Although none of the regression models was significant, we still examined significant predictors relevant to our hypotheses.

First, there was a significant WM × TAI interaction on the testing effect for boring facts in delayed test. To examine the relationship between trait test anxiety and testing effect as a function of participants’ WM capacity, we divided participants into high vs. low WM groups by median split (*N* = 47 and 49 for high vs. low groups), based on their WM scores (high: *M* = 58.83, *SD* = 7.44; low: *M* = 34.12, and *SD* = 9.06) and performed multiple regression analyses on each group.^3^ The TAI scores were statistically equivalent for high (*M* = 43.64, *SD* = 9.68) vs. low WM group (*M* = 46.92, *SD* = 11.48), *t*(94) = 1.51, *p* = 0.13. The simple main effect of TAI was significantly negative for low-WM participants, β = -0.37, *t*(44) = 2.41, and *p* = 0.02, but non-significant for high-WM participants, β = +0.23, *t*(42) = 1.52, and *p* = 0.14. The low-WM participants showed a weaker testing effect in delayed test when they were higher in trait test anxiety, consistent with Hypothesis 3 (i.e., Tse and Pu, 2012), although this result only occurred for boring facts.

Second, we obtained a significant three-way interaction on the testing effect for interesting facts in immediate test. Follow-up analyses showed a significant WM × TAI interaction in the full attention condition, β = +0.54, *t*(43) = 3.87, and *p* < 0.01, but not in the divided attention condition, β = -0.25, *t*(43) = 1.50, and *p* = 0.14. We then divided participants in the full-attention condition into high vs. low WM groups by median split (*N* = 25 and 23 for high vs. low groups), based on their WM scores (high: *M* = 62.32, *SD* = 7.98; low: *M* = 36.96, and *SD* = 8.67) and ran multiple regression analyses on each group.^4^ The TAI scores were statistically equivalent for low (*M* = 42.78, *SD* = 10.36) vs. high WM group (*M* = 44.60, *SD* = 10.25), *t*(46) = 0.61, and *p* = 0.54. The simple main effect of TAI was significantly positive for high-WM participants, β = +0.51, *t*(22) = 2.82, and *p* = 0.01, but non-significant for low-WM participants, β = -0.23, *t*(20) = 1.06, and *p* = 0.30. The result that high-WM participants showed stronger testing effect for interesting facts in immediate test when they were higher in trait test anxiety was not in line with Hypothesis 3.^5^

## Experiment 2: Effect of Induced Neutral Vs. Anxious Mood on Test-Enhanced Learning

### Methods

#### Sample Size Determination

Given that no prior study has directly examined the effect of anxious vs. neutral mood on test-enhanced learning, we decided to recruit the same sample size as in Experiment 1.

#### Participants and Design

A 2 (practice mode, within participants: restudying or testing) × 2 (mood, between participants: anxious or neutral) mixed-factor design was used. Forty-eight participants were recruited for each between-subject condition (see Table 3 for their age and male: female ratio information).

**Table 3:** Experiment 2’s cell means and standard deviation (in parentheses).

	Neutral mood	Anxious mood
Participants’ age	19.98 (1.41)	19.65 (1.64)
Participants’ male : female ratio	21:27	21:27
Proportion correct for interesting facts in the…		
Testing condition in the first/second practice cycle	0.66 (0.19)/0.88 (0.12)	0.67 (0.12)/0.91 (0.08)
Restudying/Testing condition in the immediate test	0.92 (0.08)/0.95 (0.08)	0.92 (0.09)/0.96 (0.06)
Restudying/Testing condition in the delayed test	0.89 (0.10)/0.93 (0.09)	0.92 (0.09)/0.94 (0.07)
Proportion correct for boring facts in the…		
Testing condition in the first/second practice cycle	0.34 (0.17)/0.68 (0.19)	0.42 (0.21)/0.75 (0.18)
Restudying/Testing condition in the immediate test	0.76 (0.16)/0.84 (0.14)	0.76 (0.15)/0.88 (0.13)
Restudying/Testing condition in the delayed test	0.76 (0.18)/0.80 (0.12)	0.77 (0.17)/0.86 (0.15)
WM score	41.44 (14.50)	51.27 (15.80)
TAI score	47.54 (12.98)	45.52 (11.08)
STAI-S positive/negative subscale…		
Before the mood induction	25.85 (6.33)/17.65 (5.88)	25.25 (5.71)/18.00 (6.23)
After the mood induction	25.54 (6.88)/16.56 (5.45)	18.69 (6.75)/21.67 (6.69)
At the end of practice cycles	26.90 (6.55)/14.92 (4.44)	25.58 (5.95)/17.12 (5.88)
At the beginning of final test	27.75 (6.59)/15.21 (5.77)	28.00 (6.52)/15.81 (6.23)
At the end of final test	28.81 (6.38)/14.79 (4.67)	28.71 (5.29)/14.94 (5.42)


### Materials

To ensure that the picture stimuli were selected based on how they were felt of in the current student population, we performed a norming task, in which 20 participants were asked to rate, on 9-point scales, valence, arousal, sadness, and anxiety of 610 pictures from International Affective Picture Stimuli database. We eliminated pictures that are too frightening (e.g., mutilated faces) or disgusting (e.g., feces) and asked the participants to distribute their ratings as evenly as possible. Based on these ratings, 120 neutral and 120 negative pictures were chosen (see Table 4). Following previous studies (e.g., Perkins et al., 2008), we used music clips “Wind on Water” by Fripp and Eno and “Solitude” by Sakamoto for the neutral mood condition and music clips “Dies irae, dies illa” by Verdi and “Battle On the Ice” by Prokofiev for the anxious mood condition. All four clips were truncated to about 5 minutes long. Each neutral/anxious music clip was paired with 60 neutral/anxious pictures, each presented for 5 s. The pairings of two neutral/anxious music clips and two sets of 60 neutral/anxious pictures were counterbalanced between participants. This music + picture mood induction procedure was reportedly the most effective way to induce participants’ negative mood (e.g., Zhang et al., 2014).

**Table 4:** Characteristics of neutral and negative pictures in Experiment 2.

	Inter-rater reliability (in Cronbach’s α)	Neutral Pictures	Negative Pictures	*p*
Anxiety	0.85	3.73 (0.48)	6.60 (0.39)	<0.01
Valence	0.88	4.98 (0.33)	2.94 (0.40)	<0.01
Arousal	0.81	3.92 (0.59)	6.38 (0.43)	<0.01
Sadness	0.84	5.03 (0.51)	3.30 (0.50)	<0.01


**The p column indicates the significance level of the difference between the negative pictures and neutral pictures.**

### Procedures

The procedure followed the same as in General Method except the following changes. Immediately following the study phase, no color-judgment filler task was given. Instead, all participants filled out STAI-S that measured their state anxiety prior to the mood induction. Then, participants in the neutral/anxious mood condition were asked to see a series of neutral/anxious pictures with the neutral/anxious music clip being played in the background. Following previous studies (e.g., Zhang et al., 2014), all participants were instructed to use their imagination to make the images more personal and to allow themselves to be carried into a deeper affective state. Following the mood induction phase, participants again filled out STAI-S, which provided the manipulation check on whether the mood induction triggered expected mood states. Then, they received the first cycle of practice phase. To boost the effect of mood induction, we had participants go through another set of music + picture neutral/anxious mood induction stimuli after the first and before the second practice cycle. At the end of practice phase and before the immediate test, all participants filled out STAI-S once again. In the delayed test after 2 days, participants filled out STAI-S before and after the test. These measured the test-induced state anxiety that might modulate the testing effect—an issue to our knowledge has not been explored in previous studies.

### Results

The WM and TAI scores were not correlated (*r* = +0.02, *p* = 0.87). The cell means are reported in Table 3. As there was a significant difference in WM scores between participants in the neutral and anxious mood conditions, *t*(94) = 3.18, *p* < 0.01, and *d* = 0.65, but not in TAI scores, *t*(94) = 0.82, *p* = 0.41, and *d* = 0.17, the WM score was included as a covariate in the following analyses. The testing performance in the first practice cycle in the two conditions differed in boring facts, *t*(94) = 2.12, *p* < 0.05, and *d* = 0.43, but not in interesting facts, *t*(94) = 0.35, *p* = 0.73, and *d* = 0.07. Their testing performance in the second practice cycle differed marginally in boring facts, *t*(94) = 1.83, *p* = 0.07, and *d* = 0.37, but not in interesting facts, *t*(94) = 1.45, *p* = 0.15, and *d* = 0.30.

Participants’ scores in positive and negative subscales in the practice cycles were separately submitted to a 3 (time point) × 2 (mood) mixed-factor ANCOVA. Controlling for WM scores, there was a time point × mood interaction in positive and negative subscale scores of STAI-S, *F*(2,186) = 13.29, *MSE* = 17.76, *p* < 0.01, $\eta_{p}^{2}$ = 0.13 and *F*(2,186) = 3.13, *MSE* = 13.31, *p* < 0.05, $\eta_{p}^{2}$ = 0.03, respectively. For positive subscale scores, participants in the neutral mood condition was significantly more positive than those in the anxious mood condition after mood induction, *F*(1,93) = 20.23, *MSE* = 46.78, *p* < 0.01, and $\eta_{p}^{2}$ = 0.18, but the scores of the two conditions did not differ before mood induction, *F*(1,93) = 0.21, *MSE* = 36.72, *p* = 0.65, and $\eta_{p}^{2}$ = 0.002 or at the end of practice cycles, *F*(1,93) = 0.84, *MSE* = 39.56, *p* = 0.36, and $\eta_{p}^{2}$ = 0.009. More critical to the purpose of our mood induction manipulation, for negative subscale scores participants in the anxious mood condition was significantly more negative than those in the neutral mood condition after mood induction, *F*(1,93) = 12.77, *MSE* = 37.16, *p* < 0.01, and $\eta_{p}^{2}$ = 0.12, and at the end of practice phase, *F*(1,93) = 5.55, *MSE* = 26.98, *p* = 0.02, and $\eta_{p}^{2}$ = 0.06. The scores of the two conditions did not differ before mood induction, *F*(1,93) = 0.15, *MSE* = 37.08, *p* = 0.70, and $\eta_{p}^{2}$ = 0.002. This shows the effectiveness of mood induction manipulation in the practice phase. The 2 (time point) × 2 (mood) interaction was not significant in positive and negative subscale scores, *F*(1,93) = 0.44, *MSE* = 8.17, *p* = 0.51, $\eta_{p}^{2}$ = 0.005 and *F*(1,93) = 0.19, *MSE* = 8.38, *p* = 0.66, $\eta_{p}^{2}$ = 0.002, respectively, suggesting that positive and negative mood did not significantly differ between the neutral and anxious mood conditions before and after the final test.

#### Does Anxious Mood Modulate the Testing Effect?

**FIGURE 2:**
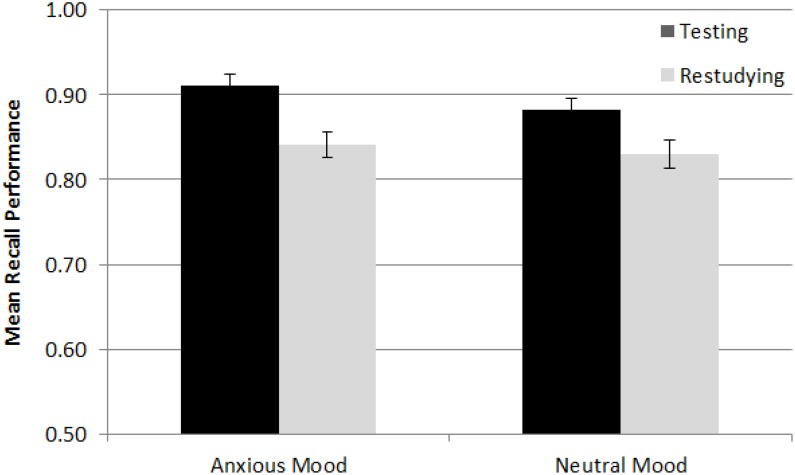
Mean recall performance in the testing and restudying condition of the neutral and anxious mood conditions in Experiment 2. The cell means were averaged across retention intervals (immediate and delayed) and across item types (interesting and boring facts). Error bars indicate the standard errors of means.

Participants’ mean proportion correct in the final tests was submitted to a 2 (practice mode) × 2 (retention interval) × 2 (item type) × 2 (mood) mixed-factor ANCOVA (see “Appendix C” for all statistics). All but mood were within-subject variables, with participants’ WM score being included as a covariate. The main effect of item type suggested that participants’ overall performance was better for interesting facts (0.93) than boring facts (0.80) [*F*(1,93) = 17.03, *MSE* = 0.02, *p* < 0.01, and $\eta_{p}^{2}$ = 0.16]. The main effect of practice mode showed an overall testing effect (0.06) [*F*(1,93) = 22.57, *MSE* = 0.01, *p* < 0.01, and $\eta_{p}^{2}$ = 0.20]. The practice mode × retention interval interaction showed that the testing effect was stronger in the immediate test (0.07) than in the delayed test (0.05) [*F*(1,93) = 4.17, *MSE* = 0.003, *p* = 0.04, and $\eta_{p}^{2}$ = 0.04]. As indicated by the marginal item type × practice mode interaction, the testing effect was stronger for boring facts (0.09) than interesting facts (0.04) [*F*(1,93) = 3.74, *MSE* = 0.01, *p* = 0.06, and $\eta_{p}^{2}$ = 0.04]. This result was consistent with Experiment 1’s one, in which participants showed a stronger testing effect for boring facts than interesting facts. Finally, there was no significant mood × practice mode interaction [*F*(1,93) = 3.66, *MSE* = 0.01, *p* = 0.06, and $\eta_{p}^{2}$ = 0.04] (see Figure 2). Furthermore, the pattern was opposite to Hypothesis 2: the testing effect was marginally weaker in the neutral mood (0.05) than anxious mood condition (0.07). Given that there was no precedence, the remaining marginal high-order interactions (see “Appendix C”) were not considered further.

#### Do Individual Difference Factors Modulate the Influence of Anxious Mood on the Testing Effect?

We first examined whether participants’ trait test anxiety in the anxious mood condition was correlated with the change in their self-rated negative mood before and after the mood induction. Across 48 participants in this condition, those who reported higher trait test anxiety did show a larger increase in self-rated negative mood before and after the mood induction, *r* = +0.32, *p* = 0.03. This correlation did not occur in the neutral mood condition, *r* = -0.10, *p* = 0.49. Next, we tested whether participants’ trait test anxiety and WM capacity could modulate the effect of experimentally induced anxious mood on the benefit of testing (relative to restudying) in multiple regression analyses. The mood variable was coded as +0.5 for neutral mood and -0.5 for anxious mood condition. All procedure followed Experiment 1 except replacing the attention variable with the mood variable. There was no problem of multicollinearity, as indicated by a high tolerance (all>0.69). The results of the full regression models are listed in “Appendix D.” There was a significant main effect of WM for interesting facts in immediate test, which was modulated by TAI. We then divided participants into high (*M* = 59.88, *SD* = 7.93) vs. low (*M* = 32.83, *SD* = 8.54) WM groups by median split (*N* = 48 and 48 for high vs. low groups) and performed multiple regression analyses on each group. The TAI scores were statistically equivalent for low-WM (*M* = 44.50, *SD* = 11.29) vs. high-WM participants (*M* = 48.56, *SD* = 12.55), *t*(94) = 1.67, *p* = 0.10. The simple main effect of TAI was significantly positive for low-WM participants, β = +0.46, *t*(43) = 2.59, *p* = 0.01, but not significant for high-WM participants, β = -0.09, *t*(43) = 0.52, *p* = 0.61. The low-WM participants showed a stronger testing effect for interesting facts in immediate test when they were higher in trait test anxiety, inconsistent with Hypothesis 3. The absence of TAI × mood interaction on the testing effect in immediate and delayed tests for interesting and boring facts suggested that even though participants with higher trait test anxiety were more likely to show an increase in self-rated negative mood in the anxious mood condition, they were not susceptible to greater influence of experimentally induced anxious mood on the testing effect.^6^

## Discussion

The benefit of testing over restudying on memory has been reported (e.g., Putnam et al., 2016). However, few studies have tested whether situational factors (e.g., divided attention manipulation during practice phase) and individual-difference factors (e.g., participants’ WM capacity) could modulate the testing effect. The overarching goal of the current experiments was to examine the influences of distraction (as triggered by divided attention and experimentally induced test-irrelevant anxious mood) on the testing effect for the learning of general knowledge facts. Individual difference factors, WM capacity and trait test anxiety, were also measured to examine whether they could modulate the testing effect. In Experiment 1, we examined how divided attention might affect the testing effect by introducing a secondary verbal task during practice phase. Yet we did not find a significant effect of divided vs. full attention on the testing effect, regardless of item type or retention interval, contrary to Hypothesis 1a or 1b. After restricting the analyses on the general knowledge facts that were practiced while participants correctly responded to the distracting task, we found that the testing effect was stronger in the divided than full attention condition, which was consistent with Hypothesis 1a and in line with the results of Buchin and Mulligan (2017, see also Mulligan and Picklesimer, 2016), as well as the prediction based on Santangelo and his colleagues’ works (e.g., Santangelo, 2018).

In Experiment 2, prior to and during the practice phase we had our participants see pictures and listen to background music that was found to effectively induce anxious mood in the literature (e.g., Zhang et al., 2014). The participants in the anxious mood condition reported higher STAI negative subscale scores than those in the neutral mood condition after the mood induction and at the end of practice phase, showing the effectiveness of our mood induction procedure. Nevertheless, we did not find any negative influence of anxious mood on the testing effect, inconsistent with Hypothesis 2. Neither picture nor music in our mood induction phase was related to the test situation, suggesting that the detrimental effect of anxious mood on test-enhanced learning did not occur when the mood was not triggered by experience relevant to the test situation. Similarly, Szõllõsi et al. (2017) did not find any influence of psychosocial stress on the testing effect on the learning of Swahili–Hungarian word pairs when participants were asked to prepare and then give a speech in front of judges (i.e., not related to word-pair learning) immediately before the final test. In contrast, Smith et al. (2016) showed that retrieval practice protected memories against the impact of psychosocial stress induced immediately before the final test. Agarwal et al. (2014) showed that middle and high school students reported less nervous about unit tests and exams after adopting retrieval practice as their study strategy. It appears that using retrieval practice to prepare for a test could reduce the effect of anxiety on subsequent test performance. The causal relationship between state anxiety and the benefit of testing should be further clarified in future studies.

We conducted multiple regression analyses to test whether situational factors (attention being divided or anxious mood being induced) interacted with participants’ individual difference factors (WM capacity and trait test anxiety) in their influence on the testing effect. The testing effect was not predicted by any of these situational factors or their interaction with the individual-difference factors, indicating that the null effects of divided attention or anxious mood were unlikely due to the genuine effects being masked by participants’ individual differences. Following Santangelo and Macaluso (2013), we expected that participants with smaller WM capacity might be more likely to allocate attentional resources to highly salient items (i.e., those being retrieved) than to not as salient items (i.e., those not being retrieved) under divided attention and thus more susceptible to the influence of divided attention on the testing effect. However, this was not supported by our findings that there was no WM × attention interaction on the testing effect. Moreover, we did not find a relationship between WM capacity and testing effect, consistent with the null relationship reported in some studies (e.g., Wiklund-Hörnqvist et al., 2014; Minear et al., 2018), yet contrary to the negative relationship shown in the other studies (e.g., Agarwal et al., 2016). It is noteworthy that numerous differences of manipulations across studies (e.g., presentation lag between initially studied and restudied/tested items) might cloud the direct comparison and the relationship between WM capacity and testing effect might likely be moderated by various manipulations, which should be clarified in future studies.

The absence of the interactive influence of WM capacity and trait test anxiety on testing effect contradicted Hypothesis 3. This was in contrast to Tse and Pu’s (2012) result that the testing effect decreased with trait test anxiety for participants with lower, but not higher, WM capacity. Whether or not study materials were presented in participants’ native language was a critical difference between Tse and Pu and the current study, but one could argue that other factors, such as the nature of study materials (word pairs vs. general knowledge facts), could also play a role since these two types of materials might be quite different in their ease of learning. However, the extent to which the word pairs were learned (i.e., proportion correct at the end of practice phase) in Tse and Pu (0.89, on average) was actually comparable with the degree to which the interesting facts were learned in the current study (0.89, on average) and even better than the boring facts (0.71, on average) because Tse and Pu provided participants with more study-test cycles. But the WM × TAI interaction was still not systematically observed in our two experiments. Thus, Tse and Pu’s results on the learning of vocabulary in unknown foreign language (Swahili for Chinese-English bilinguals) were not generalizable when the study materials were general knowledge facts presented in Chinese (participants’ first language).

It is worth exploring whether they could be accommodated by some existing theories of testing effect. First, the stronger testing effects in boring facts and in the divided attention condition could be attributed to more desirable difficulty (Bjork, 1994). This theory posits that since people pay more efforts in retrieving the study materials than restudying them, the availability and accessibility of these materials in the final test increases with such effort (see also Kornell et al., 2011, bifurcation account). Similar hypothesis could be applied to other situations that demand more efforts, such as a more difficult task. This hypothesis was supported by the findings that more difficult practice tests (e.g., Kang et al., 2007) yielded stronger testing effects than easier tests. Consistent with this, we found that boring facts, which were more difficult to retrieve in the practice phase as indicated by participants’ performance, yielded stronger testing effects than interesting facts. Given that (a) participants had more difficulty retrieving study materials under divided than full attention in the practice phase and (b) the presence of the distracting task might cause participants to focus more on the fact retrieval than they normally did, the desirable difficulty hypothesis might predict a stronger testing effect in the divided (vs. full) attention condition. Despite the null difference between the full and divided attention conditions in our findings, when analyses were restricted to the facts that had been practiced when participants in the divided attention condition accurately responded to the distracting task, we did find a stronger testing effect in the divided (vs. full) attention, in line with the results of Buchin and Mulligan (2017; see also Mulligan and Picklesimer, 2016. and Santangelo, 2015). This could be explained by participants’ putting more effort and engagement on fact retrieval when they correctly responded to the distracting task or allocating more attentional resources to items being retrieved (and thus highly salient), but not to items being restudied (and thus not as salient), as compared with the distracting task under divided attention condition. However, not all of our findings could be accommodated by these accounts. For example, the desirable difficulty hypothesis might have predicted that the benefit of divided (over full) attention on the testing effect would be higher for boring facts, as these facts should be more difficult to learn. This contradicts the absence of the item type × practice mode × attention interaction in our finding, although this null result should be tested for replicability in future studies.

Second, according to elaborative retrieval hypothesis (e.g., Carpenter, 2009), retrieval involves a long-term memory search for a specific target via activating a network of related concepts. Testing effect occurs because participants engage in more elaborative encoding when they perform the practice test (relative to merely restudying the items), the generation of this elaborative structure can provide multiple retrieval routes to the studied items and thus facilitate the retrieval in a final test. This effect might be more salient for interesting facts, relative to boring facts, as participants might trigger more elaboration and a greater search of semantic memory when retrieving the former facts than the latter facts. This was inconsistent with our finding that boring facts yielded a stronger testing effect than interesting facts in our experiments (One could argue that participants might try their best to elaborate more when they retrieved boring facts than interesting facts. However, the potential mediator, motivation to learn, was not measured in the current study, so this possibility could not be verified). Moreover, this hypothesis might have difficulty accommodating the absence of the effects of divided attention and anxious mood. For example, when participants’ attention was divided and/or they were induced with anxious mood in the practice phase, they were unlikely to have enough attentional resources to build up the elaborative structure for items in the testing condition during the practice phase. Hence, they were supposed to show smaller testing effect in these conditions, contrary to what we actually found. On the other hand, one could argue that the mood induction procedure might not only boost participants’ anxiety level but also their arousal level (e.g., Kuijsters et al., 2016). Given that more aroused participants might trigger more elaborative encoding during retrieval, it is possible that the negative influence of anxious mood might be counteracted by the positive influence of mood arousal, leading to a null effect of anxious mood induction manipulation on the testing effect, similar to Szõllõsi et al.’s (2017) results. This possibility should be tested in the future studies by directly measuring participants’ arousal level during the practice phase.

While these existing accounts could not fully accommodate the current findings, they were not proposed to explain the effect of attention or mood on test-enhanced learning. It is important to further test if these accounts could be modified to explain the current findings.

Before concluding the current study, it is worth considering some limitations of our experiments. First, the current manipulation of divided attention and anxious mood induction might not be effective enough to modulate the testing effect. The more test-relevant mood induction procedure, such as rearranging the word order to generate a sentence that depicts the scenario of test failure experience (e.g., Tse et al., 2005), should be used to further examine the potential influence of anxious mood induction. Second, the self-rating measure on anxious mood might not be reliable, so physiological measures, such as skin conductance, could be adapted to provide convergent evidence for the change in participants’ mood due to the mood induction procedure. Third, in the divided attention manipulation, we instructed participants to pay equal attention to both restudy/testing practice and category recognition distracting task, which might not often be the case in the real-world situation, where students know very well that they are supposed to take the restudy/testing practice more seriously than the distracting task. Hence, the detrimental influence of the concurrent distracting task might have been overestimated. Future research should test whether the effect of divided attention would exist after instructing participants to pay more attention to the restudy/testing practice. Finally, it is important to note that the inclusion of the immediate test might have reduced the differences in testing effect on the delayed test between full and divided attention conditions in Experiment 1 and between anxious and neutral mood conditions in Experiment 2. Since the immediate test for both conditions are without divided attention or anxious mood manipulation, participants in the divided attention or anxious mood conditions are basically given a chance to practice without any manipulation. The fact that participants in the anxious mood (or divided attention) condition retrieved the facts under neutral mood (or full attention) in the immediate test might have contaminated the results of the delayed test and at least partially contributed to the null effect of divided attention and anxious mood in the testing effect in that test. While we did clearly show the absence of negative influence of divided attention or anxious mood on the testing effect in the immediate test, future studies should test whether our delayed test results would be replicable after eliminating the immediate test.

To our knowledge, the current study was the first to examine both situational factor (distraction) and individual-difference factors (WM capacity and trait test anxiety) on the benefit of testing over restudying on the learning of general knowledge facts in a laboratory setting. We showed no influence of anxious mood on the testing effect, suggesting that the benefit of testing is immune to anxious mood unrelated to test experience during the practice phase. While we did not find an overall impact of divided attention on the testing effect, participants showed a stronger testing effect under divided attention than under full attention when we restricted our analyses on the study materials that were restudied or retrieved in the practice phase while participants correctly responded the distracting task. The two individual difference factors, WM capacity and trait test anxiety, did not consistently modulate the testing effect. While this might suggest that students should be encouraged to incorporate retrieval practice as an effective tool toward better learning outcomes, it is noteworthy that our experiments involved only low-stakes tests, in that we did not impose any evaluative pressure, nor was any monetary compensation given based on participants’ performance. It is important to test whether the current findings would be generalized in the situation when students are given high-stakes tests under high-evaluation pressure in classroom settings.

## Ethics Statement

This study was carried out in accordance with the recommendations of Survey and Behavioral Research Ethics Committee, The Chinese University of Hong Kong, with written informed consent from all subjects. All subjects gave written informed consent in accordance with the Declaration of Helsinki. The protocol was approved by the Survey and Behavioral Research Ethics Committee, The Chinese University of Hong Kong.

## Author Contributions

C-ST, MH-MC, and W-ST contributed to the study design. C-ST and MH-MC contributed test materials and collected the data. C-ST performed data analyses and drafted manuscript. All authors provided critical revisions on the manuscript draft and approved the final version of the manuscript.

## Conflict of Interest Statement

The authors declare that the research was conducted in the absence of any commercial or financial relationships that could be construed as a potential conflict of interest.

## Footnotes

1 Although many of these works were about memory for visual stimuli, they could still shed light on the role of divided attention in modulating the benefit of testing over restudying.

2 After we completed the data collection of Experiment 1, a testing-effect study (Buchin and Mulligan, 2017) that manipulated whether the secondary task involved material-specific or material-general distracting materials was published. Study items were word pairs, whereas distracting items were nouns in the material-specific word classification secondary task or digit in the material-general odd-digit counting secondary task. Regardless of the type of distracting items, Buchin and Mulligan found a stronger testing effect in the divided than full attention condition. However, as these researchers did not include a delayed test in their Experiment 1 (where the manipulation was the most similar to ours in the current study), it is not clear whether their findings could be generalized to a delayed test.

3 There were three participants with score of 48 in the median range of WM scores, so all were assigned to the low WM group. The results remained the same when they were all assigned to the high WM group.

4 There were two participants with score of 51 in the median range of WM scores, so all were assigned to the high WM group. The results remained the same when they were all assigned to the low WM group.

5 The scores estimated by Spielberger’s (1980) TAI could be divided into two components: emotionality and worry, which are differentiated by their temporal patterns and impact on academic performance (Zeidner, 1998). Emotionality is related to physiological symptoms that stem from arousal of the autonomic nervous system, whereas worry is related to debilitating thoughts and concerns that students have about evaluative situations. Previous studies showed that the negative relationship between anxiety and test performance was weaker in emotionality scores than in worry scores (e.g., Liebert and Morris, 1967). We computed emotionality and worry scores. The two scores were highly correlated (+0.82). We found that the results for these two scores were similar to those for the overall TAI scores, although this should be interpreted with caution because the factor structures of emotionality and worry components were not always clear cut in the TAI, with some of the items reflecting a mixture of both dimensions (e.g., Zeidner, 1998).

6 Similar to Experiment 1, we also computed emotionality and worry scores. The two scores were again highly correlated (+0.79). We found that the results for these two scores were similar to those for the overall TAI scores, except that (a) the main effect of mood (i.e., testing effect being stronger in the anxious mood than in the neutral mood condition) for boring facts in delayed test was now significant whether emotionality or worry score was used (both *ps* < 0.05) and (b) the WM × TAI interaction for interesting facts in immediate test was no longer significant whether emotionality or worry score was used (both *ps* > 0.05).

## APPENDIX

**Table A:** Results of Experiment 1’s omnibus analyses.

Source	*F*	*MSE*	*p*	$\eta_{p}^{2}$
**Item type**	**45.21**	**0.02**	**<0.01**	**0.33**
**Attention**	**9.60**	**0.08**	**<0.01**	**0.09**
**Practice mode**	**12.21**	**0.01**	**<0.01**	**0.12**
Retention interval	1.47	0.01	0.23	0.02
**Item type × attention**	**6.24**	**0.02**	**0.01**	**0.06**
**Item type × practice mode**	**6.03**	**0.01**	**0.02**	**0.06**
Item type × retention interval	<0.01	<0.01	0.95	<0.01
Attention × practice mode	1.96	0.01	0.17	0.02
Attention × retention interval	0.72	0.01	0.40	0.01
Practice mode × retention interval	0.17	<0.01	0.68	0.002
Item type × attention × practice mode	0.22	0.01	0.64	0.002
Item type × attention × retention interval	0.21	<0.01	0.65	0.002
Item type × practice mode × retention interval	0.96	<0.01	0.33	0.01
Attention × practice mode × retention interval	1.75	<0.01	0.19	0.02
Item type × attention × practice Mode × retention interval	0.83	<0.01	0.37	0.01

***df* = (1,93). The significant effects are highlighted in bold fonts.**

**Table B:** Results of regression analyses on the testing effect in Experiment 1.

	Interesting fact						Boring fact					
		
	Immediate test			Delayed test			Immediate test			Delayed test		
				
	*F*	*MSE*	*p*	*F*	*MSE*	*p*	*F*	*MSE*	*p*	*F*	*MSE*	*p*
Full regression model	1.75	0.01	0.10	0.40	0.01	0.92	1.61	0.02	0.12	1.52	0.01	0.16

	β	*t*	*p*	β	*t*	*p*	β	*t*	*p*	β	*t*	*p*

Performance in the practice phase	0.04	0.34	0.73	-0.02	-0.19	0.85	0.17	1.49	0.14	0.05	0.47	0.64
Main effect of WM score	0.03	0.30	0.76	-0.01	-0.08	0.94	-0.17	-1.52	0.13	-0.11	-0.99	0.33
Main effect of TAI score	-0.18	-1.63	0.11	-0.13	-1.11	0.27	-0.16	-1.43	0.16	-0.15	-1.34	0.18
Main effect of attention	-0.18	-1.62	0.11	-0.11	-0.97	0.33	-*0.21*	-*1.88*	*0.06*	-0.06	-0.53	0.59
The interaction of…												
WM × attention	0.09	0.84	0.41	0.02	0.14	0.89	0.13	1.19	0.24	0.07	0.68	0.50
TAI × attention	0.18	1.67	0.10	0.07	0.64	0.52	-0.11	-0.99	0.32	0.02	0.15	0.88
WM × TAI	-0.01	-0.11	0.91	-0.06	-0.52	0.60	0.14	1.28	0.20	**0.29**	**2.57**	**0.01**
WM × attention × TAI	**0.31**	**2.76**	**0.01**	0.10	0.85	0.40	0.18	1.61	0.11	0.06	0.57	0.57

**The significant effects are highlighted in bold fonts. The marginal effects are highlighted in italic fonts.**

**Table C:** Results of Experiment 2’s omnibus analyses.

Source	*F*	*MSE*	*p*	$\eta_{p}^{2}$
**Item type**	**17.03**	**0.02**	**<0.01**	**0.16**
Mood	0.46	0.08	0.35	0.01
**Practice mode**	**22.57**	**0.01**	**<0.01**	**0.20**
Retention interval	1.90	0.01	0.17	0.02
Item type × mood	0.33	0.02	0.57	0.004
*Item type × practice mode*	*3.74*	*0.01*	*0.06*	*0.04*
Item type × retention interval	0.05	0.002	0.82	0.001
*Mood × practice mode*	*3.66*	*0.01*	*0.06*	*0.04*
Mood × retention interval	0.92	0.01	0.34	0.01
**Practice mode × retention interval**	**4.17**	**0.003**	**0.04**	**0.04**
*Item type × mood × practice mode*	*2.97*	*0.01*	*0.06*	*0.03*
Item type × mood × retention interval	0.82	0.002	0.37	0.01
Item type × practice mode × retention interval	0.98	0.001	0.33	0.01
Mood × practice mode × retention interval	0.50	0.003	0.48	0.01
*Item type × mood × practice mode × retention interval*	*3.66*	*0.001*	*0.06*	*0.04*

***df* = (1,93). The significant effects are highlighted in bold fonts. The marginal effects are highlighted in italic fonts.**

**Table D:** Results of regression analyses on the testing effect in Experiment 2.

	Interesting fact						Boring fact					
		
	Immediate test			Delayed test			Immediate test			Delayed test		
				
	*F*	*MSE*	*p*	*F*	*MSE*	*p*	*F*	*MSE*	*p*	*F*	*MSE*	*p*
Full regression model	3.39	0.01	0.002	1.42	0.01	0.20	1.21	0.01	0.30	1.56	0.01	0.15

	β	*t*	*p*	β	*t*	*p*	β	*t*	*p*	β	*t*	*p*

Performance in the practice phase	**0.37**	**3.75**	**<0.01**	**0.28**	**2.65**	**0.01**	0.09	0.84	0.40	-0.09	-0.83	0.41
Main effect of WM score	-**0.26**	-**2.56**	**0.01**	0.01	0.05	0.96	-*0.21*	-*1.85*	*0.07*	-0.18	-1.62	0.11
Main effect of TAI score	0.14	1.21	0.23	0.11	0.93	0.36	0.04	0.30	0.77	-0.10	-0.83	0.41
Main effect of mood	-0.03	-0.32	0.75	0.12	1.04	0.30	-0.17	-1.48	0.14	-*0.21*	-*1.90*	*0.06*
The interaction of…												
WM × mood	-0.06	-0.56	0.58	0.06	0.54	0.59	-0.18	-1.68	0.10	0.02	0.22	0.82
TAI × mood	-0.10	-0.84	0.40	-0.15	-1.24	0.22	0.09	0.73	0.47	0.08	0.65	0.52
WM × TAI	-**0.24**	-**2.17**	**0.03**	-0.05	-0.45	0.66	-0.12	-1.01	0.32	-0.10	-0.83	0.41
WM × mood × TAI	-0.12	-1.03	0.30	-0.03	-0.22	0.83	-0.02	-0.19	0.85	-0.18	-1.50	0.14

**The significant effects are highlighted in bold fonts. The marginal effects are highlighted in italic fonts.**

## References

[B1] Agarwal P. K. D’Antonio L. Roediger H. L. McDermott K. B. McDaniel M. A. (2014). Classroom-based programs of retrieval practice reduce middle school and high school students’ test anxiety. *J. Appl. Res. Mem. Cogn.* 3 131–139. 10.1016/j.jarmac.2014.07.002

[B2] Agarwal P. K. Finley J. R. Rose N. S. Roediger H. L. (2016). Benefits from retrieval practice are greater for students with lower working memory capacity. *Memory* 25 764–771. 10.1080/09658211.2016.1220579 27531308

[B3] Bjork R. A. (1994). “Memory and metamemory considerations in the training of human beings,” in *Metacognition: Knowing About Knowing*, eds Metcalfe J. Shimamura A. (Cambridge, MA: MIT Press), 185–205.

[B4] Buchin Z. L. Mulligan N. W. (2017). The testing effect under divided attention. *J. Exp. Psychol.* 43 1934–1947. 10.1037/xlm0000427 28504527

[B5] Bundesen C. Habekost T. Kyllingsbæk S. (2011). A neural theory of visual attention and short-term memory (NTVA). *Neuropsychologia* 49 1446–1457. 10.1016/j.neuropsychologia.2010.12.006 21146554

[B6] Carpenter S. K. (2009). Cue strength as a moderator of the testing effect: The benefits of elaborative retrieval. *J. Exp. Psychol.* 35 1563–1569. 10.1037/a0017021 19857026

[B7] Cassady J. C. (2004). The influence of cognitive test anxiety across the learning-testing cycle. *Learn. Instr.* 14 569–592. 10.1016/j.learninstruc.2004.09.002

[B8] Chan J. C. K. McDermott K. B. (2007). The testing effect in recognition memory: A dual process account. *J. Exp. Psychol.* 33 431–437. 10.1037/0278-7393.33.2.431 17352622

[B9] Dudukovic N. M. DuBrow S. Wagner A. D. (2009). Attention during memory retrieval enhances future remembering. *Mem. Cogn.* 37 953–961. 10.3758/MC.37.7.953 19744935 PMC2776078

[B10] Dudukovic N. M. Gottshall J. L. Cavanaugh P. A. (2015). Diminished testing benefits in young adults with attention-deficit hyperactivity disorder. *Memory* 23 1264–1276. 10.1080/09658211.2014.977921 25385006

[B11] Fernandes M. A. Moscovitch M. (2003). Interference effects from divided attention during retrieval in younger and older adults. *Psychol. Aging* 18 219–230. 10.1037/0882-7974.18.2.219 12825772

[B12] Gaspelin N. Ruthruff E. Pashler H. (2013). Divided attention: an undesirable difficulty in memory retention. *Mem. Cogn.* 41 978–988. 10.3758/s13421-013-0326-5 23690275

[B13] Gazzaley A. Nobre A. C. (2012). Top-down modulation: bridging selective attention and working memory. *Trends Cogn. Sci.* 16 129–135. 10.1016/j.tics.2011.11.014 22209601 PMC3510782

[B14] Harris L. M. Cumming S. R. (2003). An examination of the relationship between anxiety and performance on prospective and retrospective memory tasks. *Aust. J. Psychol.* 55 51–55. 10.1080/00049530412331312874 26691304

[B15] Hinze S. R. Rapp D. N. (2014). Retrieval (sometimes) enhances learning: Performance pressure reduces the benefits of retrieval practice. *Appl. Cogn. Psychol.* 28 597–606. 10.1002/acp.3032

[B16] Holmes N. (2012). *The Book of Everything, a Visual Guide to Travel and the World.* Australia: Lonely Planet Publications.

[B17] Kang S. H. K. McDermott K. B. Roediger H. L. (2007). Test format and corrective feedback modify the effect of testing on long-term retention. *Eur. J. Cogn. Psychol.* 19 528–558. 10.1080/09541440601056620

[B18] Kensinger E. A. (2009). *Emotional Memory Across the Adult Lifespan.* New York, NY: Psychology Press.

[B19] Kliegl O. Bäuml K.-H. T. (2016). Retrieval practice can insulate items against intralist interference: evidence from the list-length effect, output interference, and retrieval-induced forgetting. *J. Exp. Psychol. Learn. Mem. Cogn.* 42 202–214. 10.1037/xlm0000172 26301963

[B20] Kornell N. Bjork R. A. Garcia M. A. (2011). Why tests appear to prevent forgetting: a distribution-based bifurcation model. *J. Mem. Lang.* 65 85–97. 10.1016/j.jml.2011.04.002

[B21] Kornell N. Vaughn K. E. (2016). How retrieval attempts affect learning: a review and synthesis. *Psychol. Learn. Motiv.* 65 183–215. 10.1016/bs.plm.2016.03.003

[B22] Kuijsters A. Redi J. A. de Ruyter B. Heynderickx I. E. J. (2016). Inducing sadness and anxiousness through visual media: measurement techniques and persistence. *Front. Psychol.* 7:1141. 10.3389/fpsyg.2016.01141 27536260 PMC4971078

[B23] Kuo T. Hirshman E. (1997). The role of distinctive perceptual information in memory: Studies of the testing effect. *J. Mem. Lang.* 36 188–201. 10.1006/jmla.1996.2486

[B24] Lakens D. (2013). Calculating and reporting effect sizes to facilitate cumulative science: a practical primer for t-tests and ANOVAs. *Front. Psychol.* 4:863. 10.3389/fpsyg.2013.00863 24324449 PMC3840331

[B25] Liebert R. M. Morris L. W. (1967). Cognitive and emotional components of test anxiety: a distinction and some initial data. *Psychol. Rep.* 20 975–978. 10.2466/pr0.1967.20.3.975 6042522

[B26] MacLeod C. M. (1990). “Mood disorders and cognition,” in *Cognitive Psychology: An International Review*, ed. Eysenck M. W. (Chichester: Wiley).

[B27] Minear M. Coane J. H. Boland S. C. Cooney L. H. Albat M. (2018). The benefits of retrieval practice depend on item difficulty and intelligence. *J. Exp. Psychol.* 44 1474–1486. 10.1037/xlm0000486 29648873

[B28] Mulligan N. W. Picklesimer M. (2016). Attention and the testing effect. *J. Exp. Psychol.* 42 938–950. 10.1037/xlm0000227 26618913

[B29] Pedale T. Santangelo V. (2015). Perceptual salience affects the contents of working memory during free-recollection of objects from natural scenes. *Front. Hum. Neurosci.* 9:60. 10.3389/fnhum.2015.00060 25741266 PMC4330792

[B30] Perkins K. A. Ciccocioppo M. Conklin C. A. Milanak M. E. Grottenthaler A. Sayette M. A. (2008). Mood influences on acute smoking responses are independent of nicotine intake and dose expectancy. *J. Abnorm. Psychol.* 117 79–93. 10.1037/0021-843X.117.1.79 18266487

[B31] Pu X. Tse C. S. (2014). The influence of intentional versus incidental retrieval practices on the role of recollection in test-enhanced learning. *Cogn. Process.* 15 55–64. 10.1007/s10339-013-0580-2 24096993

[B32] Putnam A. L. Sungkhasettee V. Roediger H. L. (2016). Optimizing learning in college: tips from cognitive psychology. *Perspect. Psychol. Sci.* 11 652–660. 10.1177/1745691616645770 27694461

[B33] Rowland C. A. (2014). The effect of testing versus restudy on retention: a meta-analytic review of the testing effect. *Psychol. Bull.* 140 1432–1463. 10.1037/a0037559 25150680

[B34] Santangelo V. (2015). Forced to remember: when memory is biased by salient information. *Behav. Brain Res.* 283 1–10. 10.1016/j.bbr.2015.01.013 25595422

[B35] Santangelo V. (2018). Large-scale brain networks supporting divided attention across spatial locations and sensory modalities. *Front. Integr. Neurosci.* 12:8. 10.3389/fnint.2018.00008 29535614 PMC5835354

[B36] Santangelo V. Macaluso E. (2013). The contribution of working memory to divided attention. *Hum. Brain Mapp.* 34 158–175. 10.1002/hbm.21430 22021081 PMC6870351

[B37] Smith A. M. Floerke V. A. Thomas A. K. (2016). Retrieval practice protects memory against acute stress. *Science* 354 1046–1048. 10.1126/science.aah5067 27885031

[B38] Sorg B. A. Whitney P. (1992). The effect of trait anxiety and situational stress on working memory capacity. *J. Res. Pers.* 26 235–241. 10.1016/0092-6566(92)90041-2 24702000

[B39] Spielberger C. D. (1980). *Test Anxiety Inventory.* Palo Alto, CA: Consulting Psychologists Press.

[B40] Spielberger C. D. Gorsuch R. Lushene R. (1970). *STAI Manual for the State-Trait Anxiety Inventory.* Palo Alto, CA: Consulting Psychologists Press.

[B41] Szõllõsi Á Keresztes A. Novák B. Szászi B. Kéri S. Racsmány M. (2017). The testing effect is preserved in stressful final testing environment. *Appl. Cogn. Psychol.* 31 615–622. 10.1002/acp.3363

[B42] Tauber S. K. Dunlosky J. Rawson K. A. Rhodes M. G. Sotzman D. M. (2013). General knowledge norms: updated and expanded from the Nelson and Narens (1980) norms. *Behav. Res. Methods* 45 1114–1143. 10.3758/s13428-012-0307-9 23344739

[B43] Tse C.-S. Balota D. A. Roediger H. L. (2010). The benefits and costs of testing on the learning of face-name pairs in healthy older adults. *Psychol. Aging* 25 833–845. 10.1037/a0019933 20718541 PMC2990807

[B44] Tse C.-S. Pu X. (2012). The effectiveness of test-enhanced learning depends on trait test anxiety and working memory capacity. *J. Exp. Psychol.* 18 253–264. 10.1037/a0029190 22774786

[B45] Tse W. S. Wu H. W. Wan K. Y. (2005). “Exploring mood responses differences to interpersonal stress versus achievement stress,” *Poster Presented at the 17th Annual Convention of American Psychological Society*, Los Angeles, CA.

[B46] Unsworth N. Heitz R. P. Schrock J. C. Engle R. W. (2005). An automated version of the operation span task. *Behav. Res. Methods* 37 498–505. 10.1080/24740527.2018.1476313 16405146

[B47] Vigneau F. Cormier S. (2008). The factor structure of the state-trait anxiety inventory: an alternative view. *J. Pers. Assess.* 90 280–285. 10.1080/00223890701885027 18444124

[B48] Wiklund-Hörnqvist C. Jonsson B. Nyberg L. (2014). Strengthening concept learning by repeated testing. *Scand. J. Psychol.* 55 10–16. 10.1111/sjop.12093 24313425 PMC4235419

[B49] Yoon C. Feinberg F. Hu P. Gutchess A. H. Hedden T. Chen H. Y. M. (2004). Category norms as a function of culture and age: comparisons of item responses to 105 categories by American and Chinese adults. *Psychol. Aging* 19 379–393. 10.1037/0882-7974.19.3.379 15382989

[B50] Yue X. D. (1996). Test anxiety and self-efficacy: levels and relationship among secondary school students in Hong Kong. *Psychologia* 39 193–202.

[B51] Zeidner M. (1998). *Test Anxiety: The State of the Art.* New York, NY: Plenum.

[B52] Zhang X. Yu H. W. Barrett L. F. (2014). How does this make you feel? A comparison of four affect induction procedures. *Front. Psychol.* 5:689. 10.3389/fpsyg.2014.00689 25071659 PMC4086046

